# Understanding How Indigenous Knowledge Contributes to Climate Change Adaptation and Resilience: A Systematic Literature Review

**DOI:** 10.1007/s00267-024-02032-x

**Published:** 2024-08-31

**Authors:** Tashi Dorji, Kinley Rinchen, Angus Morrison-Saunders, David Blake, Vicki Banham, Sonam Pelden

**Affiliations:** 1 School of Science, Edith Cowan University, Perth, WA Australia; 2 School of Arts and Humanities, Edith Cowan University, Perth, WA Australia; 3 Centre for People Place and Planet, Edith Cowan University, Perth, WA Australia; 4 Research Unit for Environmental Sciences and Management, North-West University, Potchefstroom, South Africa

**Keywords:** Indigenous knowledge, Climate resilience, Climate change, Adaptation, Mitigation, Sustainable development

## Abstract

Climate change is one of the biggest challenges facing the world today threatening societies and the future of the planet. The impacts of climate change are more severe in poor and marginalised populations like Indigenous communities where people rely heavily on their Indigenous Knowledge (IK) to adapt to the changing environment. Climate change adaptation and resilience are critical for the survival of Indigenous communities under the threat of climate change. This systematic literature review seeks to understand how IK contributes to climate change adaptation and resilience. A total of 71 papers from Scopus were analysed using the Preferred Reporting Items for Systematic Reviews and Meta-Analyses (PRISMA) method. It investigated three research questions: (i) How is IK understood in climate change studies? (ii) What kind of IK is used to address climate change and enhance adaptation and resilience? and finally, (iii) What could be done to maximise the use of IK towards enhancing climate adaptation and resilience? The study found that Indigenous people use IK to predict extreme climatic conditions, prepare for it, and live through it making use of Indigenous adaptation strategies in multiple manifestations. The solutions to maximise the benefits of IK promote two dominant themes requiring more research on IK and climate change with diverse focus areas and the need to bridge it with scientific knowledge. This review provides a starting point for such research that will draw upon IK to enhance climate adaptation and resilience towards meaningful sustainable development.

## Introduction

Climate change is the one of the biggest challenges facing the world today (Rodríguez and Alsop 2016) causing sudden and drastic changes (Arshad et al. 2018) impacting all societies (Heininen 2022), and threatening the survival of humanity (Arshad et al. 2018). The impacts of climate change are more severe and intense in poor and marginalised communities (Ara Parvin and Reazul Ahsan 2013) and many Indigenous communities are one of the most vulnerable (Leal Filho et al. 2021). Adapting to the multidimensional changes brought forth by climate change, Indigenous communities rely heavily on their Indigenous Knowledge (IK) systems to enhance adaptation and resilience to climate change (Apraku et al. 2018). IK refers to the repository of knowledge, practices, beliefs, and insights developed over generations within Indigenous communities (Sukula 2006). IK is deeply rooted in the cultural, social, and ecological contexts of specific Indigenous groups and is closely tied to their relationship with the land, the environment, and traditional ways of life (Kusumastuti et al. 2023; Radcliffe et al. 2020). The use of IK, alongside scientific knowledge provides an efficient solution to enhance climate change adaptation and build the resilience of the Indigenous communities in the face of a changing climate (Chen and Cheng 2020), which has also increased the severity and frequency of disasters (Heininen 2022).

Within the context of disaster management, climate change adaptation and resilience are studied from multiple perspectives of ecological, economic and social contexts (Mahmood et al. 2021; Shirgir et al. 2019). Adjustments within these multiple perspectives are required in response to actual or expected climate changes by modifying processes, practices, or structures to mitigate damage or exploit opportunities (Ipcc.ch, 2024). Thus, with the focus on IK, climate change adaptation involves adjusting to the physical environment or human systems in response to potential or anticipated climatic shocks and their impacts (Jiri et al. 2016; Kom et al. 2023; Taylor et al. 2023). Resilience, on the other hand, is a multidimensional phenomenon with varying definitions and interpretations based on purpose, context, scale, discipline, domain, and system (Rana 2020). Climate resilience refers to “the ability of a system and its component parts to anticipate, absorb, accommodate, or recover from the effects of a hazardous event in a timely and efficient manner,” (IPCC, 2012, p. 5), or the ability of individuals and communities to withstand, adapt to, and recover from the adverse impacts of climate change (Ngare et al. 2022; Mahmood et al. 2021; Shirgir et al. 2019). The frameworks of climate change adaptation and resilience are critically important, as they permeate various domains such as disaster risk reduction, climate adaptation strategies, and broader sustainable development efforts, highlighting their fundamental role in addressing complex global challenges (Rana 2020).

This paper provides the results of a systematic literature review (SLR) of global literature to understand how IK contributes to climate change adaptation and climate resilience. Three research questions were explored:

- How is IK understood in climate change studies?
- What kind of IK is used to address climate change and enhance adaptation and resilience?
- What could be done to maximise the use of IK towards enhancing climate adaptation and resilience?

## Methodology

**Fig. 1:**
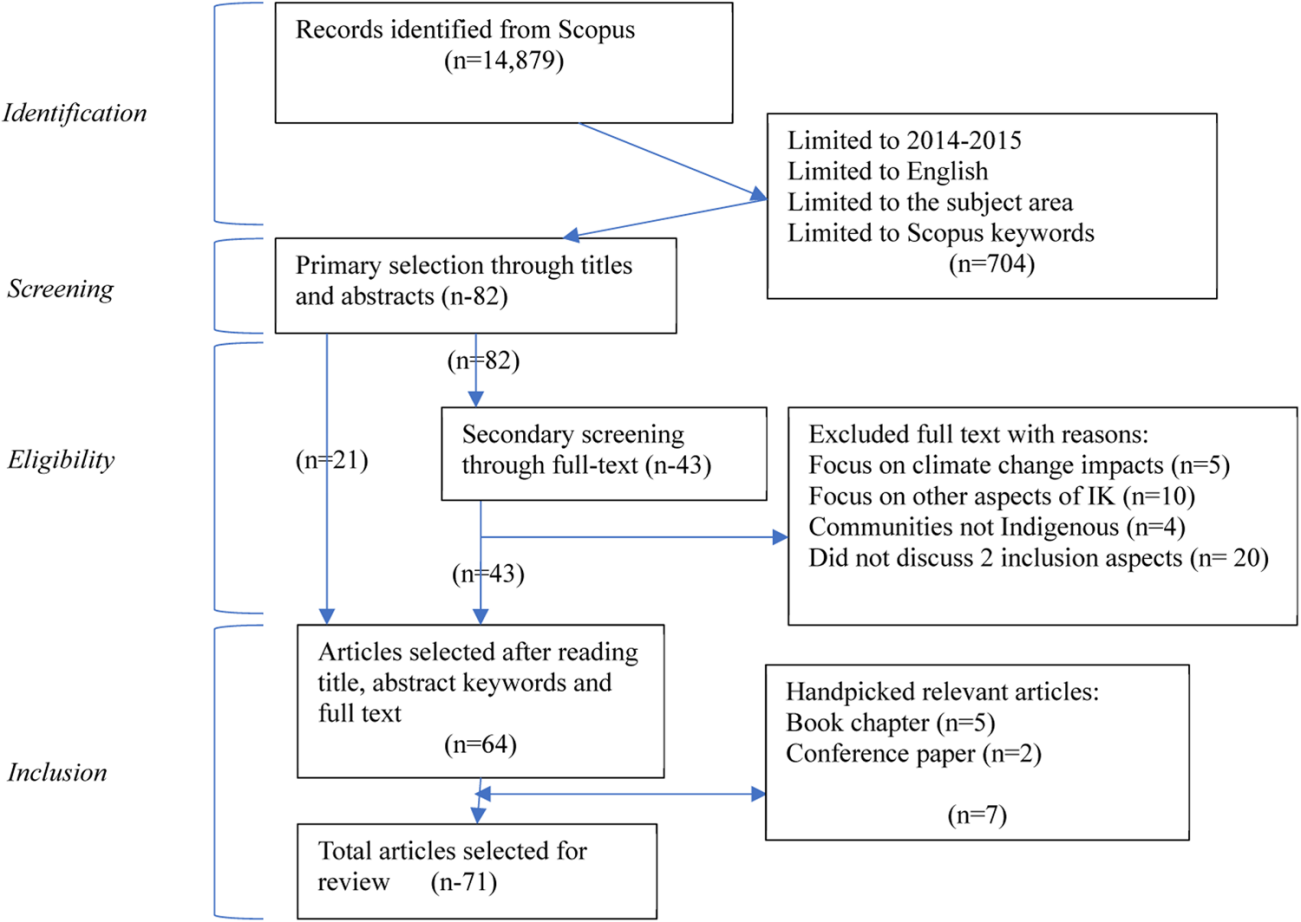
PRISMA flow diagram showing review process adapted from Legide et al. (2024)

A SLR is considered credible with rigorous methodological approaches (Xiao and Watson 2019) answering specific research questions with critical evaluation of study results (Rother 2007). The Preferred Reporting Items for Systematic Reviews and Meta-Analyses guidelines (Sarkis-Onofre et al. 2021) is used, which entails a systematic progression of four stages of identification, screening, eligibility assessment, and inclusion of literature (as shown in Fig. 1) for final analysis (Page et al. 2021). A search protocol was used to identify suitable literature, and a set of inclusion/exclusion criteria were used to narrow down the papers to the research topic, which is followed by titles and abstracts, and full-text reading (Page et al. 2021; Sarkis-Onofre et al. 2021).

Literature was searched in Scopus database, which is one of the most popular, credible, and used bibliographic databases (Pranckutė 2021; Mongeon and Adèle Paul-Hus 2015) and is also the “largest single abstract and indexing database ever built” (Burnham 2006, p. 1). A comprehensive search to identify literature on IK and climate adaptation and resilience was conducted using the search keywords: ((Indigenous OR traditional OR local OR aboriginal OR tribal OR native) AND (knowledge* OR practi?e* OR approach*) AND (climate OR “climate change” OR “global warming” OR “chang* climate”) AND (resilience* OR adapt*)) in the title, abstract, and keywords (published to May 2024). The search yielded 14,879 papers.

The inclusion/exclusion criteria used are based on the SLR by Taylor et al. (2023) which focused on climate change adaptation trends among Indigenous peoples and found that there is an increasing number of research on the topic in the past 20 years with the highest number of journal articles published between 2017 and 2019. Therefore, publications are limited to those published since 2014 for currency like Taylor et al. (2023), peer-reviewed journal articles and reviews in the English language. This reduced the number of eligible papers to 9668. To improve the relevance of the papers to the topic under investigation, the subject area (Burnham 2006) was limited to environment sciences, social sciences, agriculture and biological sciences, earth and planetary sciences, arts and humanities, and multidisciplinary, which further reduced the number of papers to 8899.

The option provided by Scopus to limit the selected papers by their indexed keywords (Singh et al. 2021) was used to reduce the number of papers even further. Scopus indexed keywords are generated from author keywords (Lu et al. 2021), which are a list of topic-specific words chosen by authors that gives information about the topics under investigation (Sun and Teichert 2022; Zhang et al. 2015), and summarises and characterises the content of the scientific publication (Kwon, 2018). Keywords contain important information for both human indexing and automatic indexing systems to organise information more effectively (Fadlalla and Amani 2015). Lu et al. (2020) found that on aggregate, there is a low occurrence of author-selected keywords within the titles and abstracts, which adversely affects the precision of automatic keyword extraction techniques. Following the guidance of Lu et al. (2020) to refine the papers to have a specific emphasis on IK, the papers were limited to keyword variants of IK provided by Scopus comprising traditional knowledge, Indigenous knowledge, Indigenous population, and local knowledge, and it reduced the paper pool to 704.

The next step was a review of titles and abstracts which included 21 papers and selected 82 more for full-text reading. The final inclusion criteria employed were based on; (i) how IK was used to address climate change, and (ii) how the usage of IK contributed to adaptation and resilience. It became necessary for papers to discuss both the above aspects to effectively meet the selection criteria. This also meant that papers not specifically discussing IK and climate change adaptation or resilience were excluded like those with exclusive focus on climate change impacts, other aspects of IK, communities that are not Indigenous, emission reduction etc. This reduced the final paper number to 64. After the initial selection, the authors pooled in 7 peer-reviewed book chapters and conference papers found during their investigation and it was screened for the inclusion/exclusion criteria taking the total papers in the final suite to 71.

An analysis framework of Fatoric and Seekamp (2017) used by Dorji et al. (2023) was adopted to guide the analysis of the final selected papers. This included studying the general publication characteristics of the 71 final papers offering global contextual trends on the research topic. Using NVivo (Faber et al. 2019) for data analysis, the coding process began with open coding which noted different ideas and concepts that were guided by the research questions. As patterns started emerging, codes were assigned to the themes that were further refined as coding progressed (Faber et al. 2019). Finally, a thematic analysis was conducted to systematically identify and interpret patterns within the data and the findings of this paper are presented according to the themes that emerged during the coding process Table 1.

**Table 1:** The Analysis Framework Questionnaire Adapted from Dorji et al. (2023)

General Publication characteristics	• Publication period • Authorship • Publication type and details • Geographical focus
Focus and Content (Research Questions)	1. How is IK understood in climate change studies? 2. What kind of IK is used to address climate change and enhance adaptation and resilience? 3. What could be done to maximise the use of IK towards enhancing climate adaptation and resilience?

## Results and Discussion

The general characteristics of the final 71 publications are outlined in Table 2. The papers are organised according to the geographical focus. The largest number of papers (*n* = 16, 23%) were published in 2021, followed by (*n* = 9, 13) in 2023 and (*n* = 8, 11%) in 2022. The lowest number was in 2014 (*n* = 3, 4%) and the number increased nominally before it slightly decreased in 2020 (*n* = 4, 6%). The increasing trend since 2014 suggests that the academic focus on the study of IK and its impact on climate change adaptation and resilience has gained momentum in recent years.

**Table 2:** General Characteristics of the 71 Papers Identified in the Systematic Literature Review (Ordered by Geography)

Item	Author(s)	Year	Title of work	Publication type and details	Geographical focus	Community focus
1.	W. L. Filho, J. Barbir, J. Gwenzi, D. Ayal, N. P. Simpson, L. Adeleke, B. Tilahun, I. Chirisa, S. F. Gbedemah, D. M. Nzengya, A. Sharifi, T. Theodory, and S. Yaffa	2022	The role of Indigenous knowledge in climate change adaptation in Africa	Journal article: *Environmental Science and Policy*	Africa	19 countries in Africa
2.	W. L. Filho, F. Wolf, E. Totin, L. Zvobgo, N. P. Simpson, K. Musiyiwa, J. W. Kalangu, M. Sanni, I. Adelekan, J. Efitre, F. K. Donkor, A. Balogun, S. A. R. Mucova, D. Y. Ayal	2023	Is Indigenous knowledge serving climate adaptation? Evidence from various African regions	Journal article: *Development Policy Review*	Africa	Multiple countries in Africa
3.	Y. Y. Legide, G. S. Feyissa and T. M. Karo	2024	Revitalising Indigenous practices employed by farmers to reduce agriculture’s vulnerability to climate change: a systematic review	Journal article: *Journal of Environmental Studies and Sciences*	Africa	Indigenous communities in Africa
4.	Z. Y. Amare	2018	Indigenous knowledge of Rural Communities for Combating Climate Change Impacts in West Central Ethiopia	Journal article: *Journal of Agricultural Extension*	Ethiopia	Dejen district of west-central Ethiopia
5.	L. Guodaar, D. K. Bardsley and J. Suh	2021	Indigenous adaptation to climate change risks in northern Ghana	Journal article: *Climatic Change*	Ghana	6 communities in three districts of northern regions of Ghana
6.	L. A. Napogbong, A. Ahmed and E. K. Derbile	2021	Fulani herders and Indigenous strategies of climate change adaptation in Kpongu community, North-Western Ghana: implications for adaptation planning	Journal article: *Climate and Development*	Ghana	Kpongu community
7.	S. Opare	2018	Adaptation to climate change impacts: coping strategies of an Indigenous community in Ghana to declining water supply	Journal article: *Climate and Development*	Ghana	Dupong in Eastern Ghana.
8.	H. Mensah, D. K. Ahadzie, S. A. Takyi, & O. Amponsah	2021	Climate change resilience: lessons from local climate-smart agricultural practices in Ghana	Journal article: *Energy, Ecology and Environment*	Ghana	Eight farming communities in West Ghana
9.	F. Mwaniki and R. B. Stevenson	2017	Farmers’ uses of Indigenous knowledge and practices to cope with climate change in KilifiCounty, Kenya	Journal article: *International Journal of Climate Change: Impacts and Responses*	Kenya	KilifiCounty
10.	Wekesa, L. Ndalilo, P. Ongugo, N. Leley, & K. Swiderska	2015	Traditional knowledge-based innovations for adaptation and resilience to climate change: the case of coastal Kenya	Conference paper: *XIV World Forestry Congress, Durban, South Africa, 7-11 September, 2015*	Kenya	Five farming communities in coastal Kenya
11.	A Apraku, J. F. Morton, and B. A. Gyampoh	2021	Climate change and small-scale agriculture in Africa: Does Indigenous knowledge matter? Insights from Kenya and South Africa	Journal article: *Scientific African*	Kenya and South Africa	Kisumu County in Kenya and Eastern Cape Province in South Africa
12.	E. C. Nkomwa, M. K. Joshua, C. Ngongondo, M. Monjerezi and F. Chipungu	2014	Assessing Indigenous knowledge systems and climate change adaptation strategies in agriculture: A case study of Chagaka Village, Chikhwawa, Southern Malawi	Journal article: *Physics and Chemistry of the Earth*	Malawi	Chagaka Village in Mbewe Extension Planning Area, Chikhwawa district, Southern Malawi
13.	M. E. Fernández-Giménez, A. El Aich, O. El Aouni, I. Adrane and S. El Aayadi	2021	Ilemchane Transhumant Pastoralists’ Traditional Ecological Knowledge and Adaptive Strategies: Continuity and Change in Morocco’s High Atlas Mountains	Journal article: *Mountain Research and Development*	Morocco	High Atlas Mountains
14.	A. Matos, L. Barraza and I. Ruiz-Mallén	2021	Linking conservation, community knowledge, and adaptation to extreme climatic events: A case study in Gorongosa National Park, Mozambique	Journal article: *Sustainability (Switzerland)*	Mozambique	Muanadimae and Nhanfisse communities at Gorongosa National Park
15.	L. J. Hooli	2016	Resilience of the poorest: coping strategies and Indigenous knowledge of living with the floods in Northern Namibia	Journal article: *Regional Environmental Change*	Namibia	Four administrative regions of Ohangwena, Omusati, Oshana, and Oshikoto along the Cuvelai-Etosha River basin
16.	M. W. Musa & S. Umar	2017	Advancing the Resilience of Rural People to Climate Change through Indigenous Best Practices: Experience from Northern Nigeria	Book chapter: *Climate Change Management*	Nigeria	Two agro-ecological communities of Katsina State
17.	N. K. Taremwa, M-C. Gasingirwa & D. Nsabimana	2022	Unleashing traditional ecological knowledge for biodiversity conservation and resilience to climate change in Rwanda	Journal article: *African Journal of Science Technology Innovation & Development*	Rwanda	Local community around Nyungwe National Park
18.	Z. Kom, N. S. Nethengwe, S. Mpandeli, & H. Chikoore	2023	Indigenous knowledge indicators employed by farmers for adaptation to climate change in rural South Africa	Journal of Environmental Planning and Management	South Africa	Levubu and Nwanedi communities
19.	K. R. Kaganzi, A. Cuni-Sanchez, F. McHarazo, E. H. Martin, R. A. Marchant and J. P. R. Thorn	2021	Local perceptions of climate change and adaptation responses from two mountain regions in Tanzania	Journal article: *Land*	Tanzania	Two regions of Mount Kilimanjaro and Udzungwa Mountains
20.	T. F. Theodory	2021	Understanding the relevance of Indigenous knowledge on climate change adaptation among mixed farmers in the Ngono River Basin, Tanzania	Journal article: *African Journal of Science, Technology, Innovation and Development*	Tanzania	Farming communities in the Ngono River Basin in Missenyi District
21.	M. Nkuba, R. Chanda, G. Mmopelwa, E. Kato, M. N. Mangheni and D. Lesolle	2019	The effect of climate information in pastoralists’ adaptation to climate change: A case study of Rwenzori region, Western Uganda	Journal article: *International Journal of Climate Change Strategies and Management*	Uganda	Rwenzori region, Western Uganda
22.	R. Zougmoré, A. C. Segnon and P. Thornton	2023	Harnessing Indigenous knowledge and practices for effective adaptation in the Sahel	Journal article: *Current Opinion in Environmental Sustainability*	West Africa	Local communities in the Sahel
23.	C. C. Makondo and D. S. G. Thomas	2018	Climate change adaptation: Linking Indigenous knowledge with western science for effective adaptation	Journal article: *Environmental Science and Policy*	Zambia	Central and southern provinces of rural Zambia
24.	S. C. Sakapaji	2021	Advancing Local Ecological Knowledge-Based Practices for Climate Change Adaptation, Resilience-Building, and Sustainability in Agriculture: A Case Study of Central and Southern Zambia	Journal article: *International Journal of Climate Change: Impacts and Responses*	Zambia	Farming communities in central and southern Zambia
25.	O. Jiri, P. L. Mafongoya and P. Chivenge	2015	Indigenous knowledge systems, seasonal ‘quality’ and climate change adaptation in Zimbabwe	Journal article: *Climate Research*	Zimbabwe	Chiredzi District in Masvingo Province
26.	O. L. Kupika, E. Gandiwa, G. Nhamo, & S. Kativu	2019	Local Ecological Knowledge on Climate Change and Ecosystem-Based Adaptation Strategies Promote Resilience in the Middle Zambezi Biosphere Reserve, Zimbabwe	Journal article: *Scientifica*	Zimbabwe	Local communities around Zambezi Biosphere Reserve (MZBR)
27.	P. Mapfumo, F. Mtambanengwe and R. Chikowo	2016	Building on Indigenous knowledge to strengthen the capacity of smallholder farming communities to adapt to climate change and variability in southern Africa	Journal article: *Climate and Development*	Zimbabwe	Makoni and Hwedza districts
28.	L. Zvobgo, P. Johnston, O. M. Olagbegi, N. P. Simpson and C. H. Trisos	2023	Role of Indigenous and local knowledge in seasonal forecasts and climate adaptation: A case study of smallholder farmers in Chiredzi, Zimbabwe	Journal article: Environmental Science and Policy	Zimbabwe	Smallholder farming communities in Chiredzi
29.	H. P. Huntington, L. T. Quakenbush and M. Nelson	2017	Evaluating the Effects of Climate Change on Indigenous Marine Mammal Hunting in Northern and Western Alaska Using Traditional Knowledge	Journal article: *Frontiers in Marine Science*	Alaska	14 Native communities from Kaktovik to Mekoryuk
30.	D. Fawcett, T. Pearce, R. Notaina, J. D. Ford and P. Collings	2018	Inuit adaptability to changing environmental conditions over an 11-year period in Ulukhaktok, Northwest Territories	Journal article: *Polar Record*	Canada	The Inuit community in Ulukhaktok, Northwest Territories
31.	T. Pearce, J. Ford, A. C. Willox and B. Smit	2015	Inuit traditional ecological knowledge (TEK), subsistence hunting and adaptation to climate change in the Canadian Arctic	Journal article: *Arctic*	Canada	Inuit community in the Arctic
32.	C. A. C. Vargas, S. H. Romero & T. León-Sicard	2019	Resilience to climate variability: the role of perceptions and traditional knowledge in the Colombian Andes	Journal article: *Agroecology and Sustainable Food Systems*	Colombia	Coffee producers’ community in Colombian Andes
33.	R. Tsosie	2019	Indigenous Sustainability and Resilience to Climate Extremes: Traditional Knowledge and the Systems of Survival	Journal article: *Connecticut Law Review*	United States	No community focus
34.	R. Datta 1 and B. Kairy	2024	Decolonising Climate Change Adaptations from Indigenous Perspectives: Learning Reflections from Munda Indigenous Communities, Coastal Areas in Bangladesh	Journal article: *Sustainability*	Bangladesh	The coastal Munda Indigenous communities
35.	S. C. Sakapaji	2022	Integrating Local and Indigenous Ecological Knowledge (IEK) Systems into Climate Adaptation Policy for Resilience Building, and Sustainability in Agriculture	Journal article: *International Journal of Sustainable Development Research*	Bangladesh	Agriculture community in Barisal
36.	J. Li & F. Han	2022	Strong ethics and flexible actions, the properties of traditional ecological knowledge (TEK), as key resources for socioecological resilience to the impacts of climate change: a case study of Baojiatun, Yunnan-Guizhou Plateau karst area, southwest China	Journal article: *Ecology and Society*	China	Farming community in Baojiatun
37.	L. K. Kahlon & R. Singh	2021	Understanding Linkages Between Sustainability and Traditional Ethnoecological Knowledge (TEK): A Case Study of Paudi Bhuyans in Northern Odisha, India	Book chapter: *Climate Resilience and Environmental Sustainability Approaches: Global Lessons and Local Challenges*	India	Tribal community in Odisha
38.	P. Rao & Y. Patil	2014	Climate Resilience in Natural Ecosystems in India: Technology Adoption and the Use of Local Knowledge Processes and Systems	Book chapter: *Handbook on Climate Change Adaptation*	India	No community focus
39.	R. S. Rana, M. Kaundal, A. Katoch, S. Singh, & K. Sood	2019	Mapping Indigenous climate resilience practices in animal disease management and feed storage protection in Himachal Himalayas	Journal article: *Indian Journal of Animal Sciences*	India	Farming communities in 8 districts in Himachal Himalayas
40.	S. Inaotombi & P. C. Mahanta	2019	Pathways of socio-ecological resilience to climate change for fisheries through Indigenous Knowledge	Journal article: *Human and Ecological Risk Assessment: An International Journal*	India	Fishers’ community in Northeast India
41.	T. Ingty	2017	High mountain communities and climate change: adaptation, traditional ecological knowledge, and institutions	Journal article: *Climatic Change*	India	The pastoralist communities of Lachenpas and Dokpas in Sikkim
42.	V. S. Negi, R. K. Maikhuri, D. Pharswan, S. Thakur and P. P. Dhyani	2017	Climate change impact in the Western Himalaya: people’s perception and adaptive strategies	Journal article: *Journal of Mountain Science*	India	Mountainous part of Uttarakhand
43.	K. A. Wani and L. Ariana	2018	Impact of climate change on Indigenous people and adaptive capacity of Bajo tribe, Indonesia	Journal article: *Environmental Claims Journal*	Indonesia	Bajo tribe
44.	L. Hiwasaki, E. Luna and J. A. Marçal	2015	Local and Indigenous knowledge on climate-related hazards of coastal and small island communities in Southeast Asia	Journal article: *Climatic Change*	Indonesia, the Philippines and Timor-Leste	Indonesia, the Philippines and Timor-Leste
45.	N. Hosen, H. Nakamura and A. Hamzah	2020	Adaptation to climate change: Does traditional ecological knowledge hold the key?	Journal article: *Sustainability (Switzerland)*	Malaysia	Indigenous communities in Sarawak, Malaysian Borneo
46.	N. Tugjamba, G. Walkerden and F. Miller	2021	Adaptation strategies of nomadic herders in northeast Mongolia: climate, globalisation and traditional knowledge	Journal article: *Local Environment*	Mongolia	Ulz River Basin
47.	D. Siggia, A. Battista, & S. Tinervia	2016	The role of Indigenous Knowledge in disasters and climate change resilience. A field study in Surkhet and Dailekh districts in the mid-western region of Nepal	Conference Proceedings: *9th Annual Conference of the EuroMed Academy of Business, Warsaw, Poland, Sep, 2016*	Nepal	Local communities in Surkhet and Dailekh districts
48.	D. Upadhya, R. Dhakal, K. Khadka, S. Rana, P. Acharya, R. Rana & P. Chaudhary	2016	Local knowledge on climate-induced traits in rice for improving crop yield, food security and climate resilience	Journal article: *International Journal of Agriculture Innovations and Research*	Nepal	Rice growing community in Terai and mid-hills regions of Nepal
49.	T. K. Baul, and M. A. McDonald	2014	Agro-Biodiversity Management: Using Indigenous Knowledge to Cope with Climate Change in the Middle-Hills of Nepal	Journal article: *Agricultural Research*	Nepal	The Middle-Hills of Pokhare Khola watershed
50.	B. R. Chaudhary, G. Acciaioli, W. Erskine & P. Chaudhary	2021	Responses of the Tharu to climate change related hazards in the water sector: Indigenous perceptions, vulnerability and adaptations in the western Tarai of Nepal	Journal article: *Climate and Development*	Nepal	Two rural villages of Thapuwa and Bikri, in Bardiya district
51.	S. Rai, B. Dahal and K. C. Anup	2022	Climate change perceptions and adaptations by Indigenous Chepang community of Dhading, Nepal	Journal article: *GeoJournal*	Nepal	Dhading District in Bagmati Province of Central Nepal
52.	L. D. Landicho, R. F. Paelmo, R. D. Cabahug, C. C. de Luna, R. G. Visco and L. L. Tolentino	2016	Climate change adaptation strategies of smallholder agroforestry farmers in the Philippines	Journal article: Journal of Environmental Science and Management	Philippines	Five provinces of Silang, Cavite; Mallig, Isabela; Atok and Tublay, Benguet; and Guinobatan
53.	C. C. Launio, R. S. Batani, C. Galagal, R. Follosco and K. O. Labon	2020	Local knowledge on climate hazards, weather forecasts and adaptation strategies: Case of cool highlands in Benguet, Philippines	Journal article: *Philippine Agricultural Scientist*	Philippines	Highlands of Benguet province, north of Metro Manila
54.	R. J. Maliao, R. C. Cahilig, R. R. Cahilig & B. T. Jaspe	2022	Climate change awareness and Indigenous Knowledge Systems and Practices (IKSP) of riverine fishers in the Nabaoy River, Malay, Aklan, Philippines: linking local social capital to socio-ecological resilience amidst the changing environment and climate	Journal article: *Science Research Network Journal*	Philippines	Fisher’s community
55.	L. L. Mangada	2021	Integrating Local Knowledge in the Climate Services for Resilience: A Case of “Haiyan” Fishers	Book chapter: *Climate Change and Resilient Food Systems*	Philippines	Fisher’s community
56.	S. H. Lee and Y. J. Chen	2021	Indigenous knowledge and endogenous actions for building tribal resilience after typhoon Soudelor in northern Taiwan	Journal article: *Sustainability (Switzerland)*	Taiwan	Three Wulai tribes along Nanshi River
57.	H. N. Son, A. Kingsbury & H. T. Hoa	2021	Indigenous Knowledge and the enhancement of community resilience to climate change in the Northern Mountainous Region of Vietnam	Journal article: *Agroecology and Sustainable Food Systems*	Vietnam	Ethnic minority communities in northern Vietnam
58.	N. T. Manh and M. M. Ahmad	2021	Indigenous farmers’ perception of climate change and the use of local knowledge to adapt to climate variability: A case study of Vietnam	Journal article: *Journal of International Development*	Vietnam	Seven Indigenous groups living in Backan
59.	P. D. Nunn, R. Kumar, A. Rarai, O. Fa’anunu, T. Fong, P. Geraghty, I. Korovulavula, D. MacLaren, R. D. Plotz, L. Chambers, L. Fifita, D. Gegeo, S. McGree, H. M. Barrowman, C. Gomese, K. Cheer, M. Fong-Lomavatu, T. Heorake, E. Kekeubata, E. Kubunavanua, S. Lui, P. Malsale, S. Nemani, L. Singh-Peterson, G. Puairana, J. Rantes, and M. Waiwai	2024	Traditional knowledge for climate resilience in the Pacific Islands	Journal article: *Wiley Interdisciplinary Reviews: Climate Change*	Pacific Islands	Pacific island countries
60.	S. S. Begg, A. D. R. N’Yeurt and S. Begg	2023	Interweaving resource management with Indigenous knowledge to build community resilience in the Pacific Islands: case of the Waimanu Catchment in Viti Levu, Fiji	Journal article: *Regional Environmental Change*	Fiji	Waimanu Catchment in the island of Viti Levu
61.	M. R. U. Dean	2023	Indigenous knowledge for social cohesion, ecological and community well-being, and climate resilience: reviving Indigenous salt crafting in Vusama, Fiji	Journal article: *AlterNative*	Fiji	Vusama Village, Nadroga-Navosa Province, Viti Levu
62.	R. Clissold, K. E. McNamara, R. Westoby & V. Wichman	2023	Experiencing and responding to extreme weather: lessons from the Cook Islands	Journal article: *The International Journal of Justice and Sustainability*	Cook Islands	Communities in Cook Islands
63.	D. de Scally & B. Doberstein	2022	Local knowledge in climate change adaptation in the Cook Islands	Journal article: *Climate and Development*	Cook Islands	Cook Islands
64.	A. A. Granderson	2017	The role of traditional knowledge in building adaptive capacity for climate change: Perspectives from Vanuatu	Journal article: *Weather, Climate, and Society*	Vanuatu (Pacific Island)	Nakanamanga-speaking groups of north Tongoa
65.	A. Rarai, M. Parsons, M. Nursey-Bray and R. Crease	2022	Situating climate change adaptation within plural worlds: The role of Indigenous and local knowledge in Pentecost Island, Vanuatu	Journal article: *Environment and Planning E: Nature and Space*	Vanuatu	Pentecost Island
66.	J. Reid, E. Challies, T. M. Tau and S. Awatere	2024	Adapting to climate change through nature-based solutions and Indigenous knowledge: the case for landscape-scale ecosystem regeneration in the Rokohouia Delta	Journal article: *Kotuitui*	New Zealand	The home of Ngāi Tūāhuriri Indigenous people - Rokohouia Delta located in Aotearoa
67.	H. A. M. Shaffril, N. Ahmad, S. F. Samsuddin, A. A. Samah and M. E. Hamdan	2020	Systematic literature review on adaptation towards climate change impacts among Indigenous people in the Asia Pacific regions	Journal article: *Journal of Cleaner Production*	Asia Pacific region	Asia Pacific region
68.	E. K. Galappaththi and A. Schlingmann	2023	The sustainability assessment of Indigenous and local knowledge-based climate adaptation responses in agricultural and aquatic food systems	Journal article: *Current Opinion in Environmental Sustainability*	Global	Global Indigenous communities
69.	J. Petzold, N. Andrews, J. D. Ford, C. Hedemann and J. C. Postigo	2020	Indigenous knowledge on climate change adaptation: A global evidence map of academic literature	Journal article: *Environmental Research Letters*	Global	Peer-reviewed academic literature on Indigenous knowledge
70.	J. E. Taylor, C. Poleacovschi and M. A. Perez	2023	Climate change adaptation trends among Indigenous peoples: a systematic review of the empirical research focus over the last 2 decades	Journal article: *Mitigation and Adaptation Strategies for Global Change*	Global	Global Indigenous communities
71.	M. Sharma & R. P. Kaushik	2015	Plant genetic resources and Indigenous traditional knowledge conservation toward resilience to climate change	Book chapter: *Plant Genetic Resources and Traditional Knowledge for Food Security*	No country focus	Indigenous communities

The authorship was diverse with 224 authors for the final 71 papers. Only two papers have two first authors (Sakapaji 2021; Sakapaji 2022 and Filho et al. 2022; Filho et al. 2023). Only one has authored three papers and four others have authored two papers each. No authoritative research is observed in understanding how IK contributes to climate change adaptation and resilience. The majority (*n* = 60, 85%) of the papers are co-authored.

Of the 71 papers, 64 are journal papers, 5 book chapters and 2 conference papers. The 64 journal papers were published in 48 different journals from across the world with varying areas of focus, which clearly shows the multidisciplinary nature of IK and its impact on climate adaptation and resilience. The journal with the most papers was *Climate and Development* (*n* = 5, 8%), with four (6%) in *Environment Science and Policy* and three (5%) in *Climatic Change*. Five other journals, *International Journal of Climate Change: Impacts and Responses, Regional Environmental Change, Land, Current Opinion in Environmental Sustainability*, and *Agroecology and Sustainable Food Systems*, had two (3%) papers. None of the journals (outlined in Table 2) has an exclusive focus on IK, but instead covers a range of environmental and sustainability fields.

The geographical spread of papers is heavily skewed towards Africa (*n* = 28, 40%) and Asia (*n* = 25, 35%) besides some papers from America, Pacific Islands and three global literature reviews. Given the large and vibrant community of Indigenous people in Europe and Australia, it is surprising to note that the systematic search did not pick up any paper with an exclusive focus on the named two continents although papers with a focus on Asia Pacific region (Shaffril et al. 2020) do mention Australia. This should not be construed to mean that IK and practices are not used towards building climate change adaptation and resilience in the two continents, rather it warrants a more nuanced focus of academic research, which is a promising avenue for future research.

Findings are presented by highlighting the dominant themes first. The most prevalent theme, on the types of IK used by Indigenous communities to enhance climate adaptation and resilience, was the prediction of weather for early warning signals and disaster preparedness (*n* = 64, 90%). This was followed by the use of IK in agriculture (*n* = 52, 73%). When analysing the solutions to maximise the benefits of using IK for climate adaptation and resilience, most of the papers (*n* = 65, 92%) emphasized the need for research and bridging IK with scientific knowledge. Following this, a range of diverse options emerged. Therefore, the solutions are structured in this order.

### How is IK Understood in Climate Change Studies?

Using nomenclature and the diverse expressions used to convey the meaning of IK in climate change studies in the 71 publications studied, it was found that the majority (*n* = 54, 76%) of papers uses IK as the central theme followed by 11 papers that discuss IK as TEK or Traditional Ecological Knowledge (Fernández-Giméne et al. 2021; Hosen et al. 2020; Ingty 2017; Matos et al. 2021; Fawcett et al. 2018; Pearce et al. 2015; Li and Han 2022; Maliao et al. 2022; Tsosie 2019; Vargas 2019; Taremwa et al. 2021). One paper (Kahlon and Singh 2021) focuses on Traditional Ethnoecological Knowledge (TEK) using the same acronym. The other terms are Traditional Knowledge (Huntington et al. 2017; Nunn et al. 2024; Pearce et al. 2015; Tsosie 2019; Wekesa et al. 2015; Mangada 2021), Local Knowledge (Launio et al. 2020; Manh and Ahmad 2021; Landicho et al. 2016; Launio et al. 2020), Local Ecological Knowledge (LEK) (Sakapaji 2021; Sakapaji 2022; Kupika et al. 2019) and Sakapaji (2022) uses it synonymously with Indigenous Ecological Knowledge (IEK) and Indigenous and Local Knowledge (ILK) (Clissold et al. 2023; Filho et al. 2022; Filho et al. 2023; Galappaththi and Schlingmann 2023; Rarai et al. 2022). In literature, IK is used synonymously with ‘traditional knowledge’ and ‘local knowledge’ (de Scally and B. Doberstein 2022; Filho et al. 2023; Napogbong et al. 2021; Lee and Chen 2021; Apraku et al. 2021; Mwaniki and Stevenson 2017; Granderson 2017; Theodory 2021) folk knowledge (Granderson 2017; Dean 2023), Indigenous people’s knowledge or farmers’ knowledge (Kom et al. 2023; Dean 2023), informal knowledge or traditional science (Theodory 2021; Dean 2023), Indigenous technical knowledge, fisherman’s knowledge, or traditional wisdom (Dean 2023).

In defining IK in the context of climate change studies, the most quoted study in the suite of 71 papers is Berkes et al. (1998, 2000) with 27 (38%) papers. Using the idea of Berkes et al. (1998, 2000), IK is defined as a collection of knowledge, beliefs, practices, traditions, and customs outlining the relationships between organisms (including humans) and their environments that have evolved through adaptive processes over time and have been passed down through generations via cultural transmission (Dean 2023; Granderson 2017; Pearce et al. 2015; Rarai et al. 2022; Li and Han 2022; Maliao et al. 2022; Sharma and Kaushik 2015; Vargas 2019; Kupika et al. 2019; Mangada 2021; Wekesa et al. 2015). Indigenous practices refers to the application and utilisation of IK in real-world contexts and putting into action the skills, beliefs, and cultural practices of Indigenous peoples in various areas of life, such as agriculture, medicine, environmental management, education, and community governance. The practices are deeply embedded in the cultural heritage and environmental understanding of Indigenous communities (Apraku et al. 2021; Hooli 2016; de Scally and Doberstein 2022). Many international organisations have adopted the definitions of Berkes (Sharma and Kaushik 2015; Mangada 2021; Pearce et al. 2015; Rarai et al. 2022).

IK is often wrongly viewed as a static concept (de Scally and Doberstein 2022) of being “immutable and fixed in time and/or space” (Sharma and Kaushik 2015, p. 210) and that it will disappear in time (Sakapaji 2022). Rather, it is a dynamic concept (Sakapaji 2022; Kupika et al. 2019; Tsosie 2019; Sharma and Kaushik 2015) of knowledge of survival (Tsosie 2019) with a history of involvement with nature (Sharma and Kaushik 2015) and the potential to generate scientific knowledge and ethical norms about how humans should interact with nature (Tsosie 2019). Today, even the global academic vision has shifted from viewing IK as a static body of knowledge to a dynamic one (Sakapaji 2022) capable of adapting to new conditions (de Scally and Doberstein 2022).

The understanding of IK in climate change studies is multifaceted and dynamic, reflecting its diverse expressions and evolving nature. The majority of the 71 analysed publications underscore IK as the central theme, with Traditional Ecological Knowledge (TEK) also prominently featured. Berkes et al.’s (1998, 2000) definition, widely adopted across these studies, describes IK as a complex, adaptive system of knowledge, beliefs, and practices that have been culturally transmitted over generations. This definition has significantly influenced global organisations like WIPO, UNEP, and UNESCO. Despite misconceptions of IK as being static, contemporary scholarship increasingly recognises it as a dynamic, resilient body of knowledge crucial for survival and environmental stewardship.

### What kind of IK is used to Address Climate Change and How Does it help Enhance Climate Adaptation and Resilience?

The findings related to understanding the different kinds of IK used to address climate change and how it contributes to climate change adaptation and resilience are divided and presented in the following three sections.

#### Weather Prediction, Early Warning, and Disaster Preparedness

Indigenous people predict weather with signs of warning of impending disasters using social, bio-physical and ecological indicators (Apraku et al. 2021; Granderson 2017; Hiwasaki et al. 2015). They pay attention to behaviour and appearance of various environmental entities like insects, plants, birds and animals (Inaotombi and Mahanta 2018; Mangada 2021; Hooli 2016; Launio et al. 2020; Chaudhary et al. 2021; Apraku et al. 2021; Filho et al. 2022; Hiwasaki et al. 2015; Ingty 2017); moon-star alignment (Filho et al. 2022); animal intestine interpretation (Filho et al. 2022); and tide flow, snowdrifts, and direction of currents (Shaffril et al. 2020) and more. Such knowledge is often used to develop the traditional seasonal calendars, which consist of complex, locale-specific environmental observations. These calendars guide Indigenous people in their activities, dictate social events like ceremonies and weddings, and help predict seasons, floods, storms, big waves, and droughts (Hiwasaki et al. 2015).

Indigenous communities have different methods of forecasting disasters. For Tharu in Nepal, hens spreading their feathers under the balcony of a house is a sign of drought or flooding. To the Dejen community in Ethiopia, drought is indicated by dry fog, north-to-south dry wind, wind after rainy days (Amare 2018). Crabs moving on dry land indicate an upcoming typhoon for the highland province of Benguet in the Philippines (Launio et al. 2020). The Mirriwong people in Australia observe the blooming of Woolegalegeng flowers as a sign of an approaching thunderstorm (Shaffril et al. 2020). The fishers of Leyte Gulf in the Philippines expect typhoons and heavy rain seeing wild pigs pile up leaves and twigs to create shelter. They secure their boats to higher and safer grounds and go home to safety of impending bad weather upon seeing red skies or storms and strong winds following dark clouds (Mangada 2021).

Communities see different signs to forecast rain such as frogs croaking and a large number of low-flying dragonflies in Backan, Vietnam (Manh and Ahmad 2021); the gathering of dark clouds and ducks running around flapping their wings and bathing in the sand in KilifiCounty, Kenya, (Mwaniki and Stevenson 2017); the appearance of the Palolo sea worm (*Eunice Viridis*) in Vanuatu (Shaffril et al. 2020); thunder, lightning, dark skies, and a windy environment in Benguet, the Philippines (Launio et al. 2020); clean sky and screeching eagle for the Dejen in Ethiopia including moisturised North to South wind that signals the onset of short rainy season (Amare 2018); and chirping and wing-beating of the Jungle Owlet, croaking of frogs for an extended time in the evening and abnormal spider spinning with shorter and thicker webs in India (Inaotombi & Mahanta 2018). Similarly, halting of precipitation is indicated by the emergence of termites during the monsoon season in northeast India (Inaotombi and Mahanta 2018); and termites coming out in large numbers while raining in Backan, Vietnam (Manh and Ahmad 2021). In Sikkim in India, changing bird migratory pattern signals altered rainfall patterns (Ingty 2017).

Indigenous people often combine Indigenous knowledge with scientific information in forecasting weather and seasons like the Dejen community in Ethiopia (Amare 2018), Tharu in Nepal (Chaudhary et al. 2021), Masvingo province in Zimbabwe (Jiri et al. 2015), Inuit in Canada (Pearce et al. 2015), herders in Mongolia (Tugjamba et al. 2021), and the Sahel region in Ghana (Zougmoré et al. 2023). Indigenous communities in Ghana’s Sahel have developed knowledge-forecasting systems that are as accurate as scientific forecasts from the national meteorological department. Combining these Indigenous methods with scientific forecasting has improved reliability, usefulness, and adoption (Zougmoré et al. 2023). However, IK-based weather forecasting remains the main source of weather-related information in communities that lack science-based forecasting services, like Borana, Ethiopia (Filho et al. 2023). On the contrary, Indigenous people in Uganda don’t use IK to forecast weather because of better access to radio weather forecast data (Filho et al. 2023). Zimbabwean farmers believe that meteorological weather forecasts are too general and unreliable, but they also find their IK less reliable due to climate variability (Jiri et al. 2015). The use of IK in weather forecasting has been vital for the survival of Indigenous communities and in lessening the impacts of climate change (Shaffril et al. 2020). It enhances local adaptive capacity (Kom et al. 2023; Mwaniki and Stevenson 2017) and are widely regarded as essential resources for climate change adaptation and resilience (Filho et al. 2022).

#### Using religious and cultural beliefs towards conservation, adaptation, and resilience

Religion and belief system constitute an integral part of Indigenous tradition (Li and Han 2022; Maliao et al. 2022; Kahlon and Singh 2021; Hiwasaki et al. 2015: Matos et al. 2021) laying the foundation of the traditional system of governance and institutions (Inaotombi and Mahanta 2018: Li and Han 2022) and adaptation strategy (Theodory 2021). Indigenous people place utmost trust in God and attribute climatic change as an act of God in Kenya (Wani and Ariana 2018), communities in Asia Pacific region (Shaffril et al. 2020), Ethiopia (Amare, 2018), Cook Islands (de Scally and Doberstein 2022), and Ghana (Guodaar at al. 2021). This reliance on God and religion indicates its essential role in the value Indigenous peoples assign to IK and its incorporation into adaptation policies (de Scally and Doberstein 2022). Similarly, cultural beliefs also contribute to conservation, adaptation and resilience. The Mirriwong people in Australia strictly follow cultural beliefs in controlling harvest and hunt (Shaffril et al. 2020), which is similar to Rwanda’s restrictions on cutting down trees (Taremwa et al. 2021), and Mozambique forbidding the cutting down of some specific tree species and such practices help in strengthening social cohesion (Guodaar et al. 2021; Hiwasaki et al. 2015), building socio-ecological resilience (Matos et al. 2021; Mwaniki and Stevenson 2017), contributing to conservation (Maliao et al. 2022), adaptation and resilience strategies (Hiwasaki et al. 2015; Kahlon and Singh 2021).

#### Using IK in Agriculture Sector to Address Climate Change and Enhance Adaptation and Resilience

In the agriculture sector where IK is used to enhance climate change adaptation and resilience as applied by Indigenous farming communities, five dominant themes emerge. Here, Indigenous peoples utilise climate-smart crop varieties, readjustment of planting and harvesting time, crop rotation and intercropping, optimising agricultural methods, and traditional storage techniques. Each is explained in turn.

*Introducing climate-smart crop varieties* includes growing crops that are more resilient to seasonal variability and extreme climatic conditions (Zougmoré et al. 2023; Manh and Ahmad 2021; Shaffril et al. 2020). Farmers change crops following a poor yield in Tanzania (Kaganzi et al. 2021), or changing rainfall patterns and temperatures in the Philippines (Landicho et al. 2016). Similarly, India, Vanuatu, and Nepal also witnessed switching the main crops to alternative crops (Shaffril et al. 2020). To adapt to excessive heat, coffee farmers in the mountain communities of Tanzania grow shade trees and switch to vegetable or fruit production (Kaganzi et al. 2021). Indigenous farmers in Fiji plant yam and tapioca that survive extreme weather conditions (Shaffril et al. 2020), and Paoay highlanders of the Philippines choose crops that can endure frost and other extreme weather conditions (Launio et al. 2020). In times of flood, farmers of Bardiya district in Nepal cultivate traditional rice varieties that are more resistant to climatic conditions (Chaudhary et al. 2021), which is similar to the Naxi in China growing famine plants during periods of drought (Ingty 2017; Shaffril et al. 2020). Coastal communities also cultivate both traditional and high-yielding crop varieties for maximum yield (Wekesa et al. 2015).

*Re-adjustment of planting and harvesting time* is a common method to adapt to changing climatic conditions (Landicho et al. 2016). The Bardiya district of Nepal witnessed 90% of farmers have transitioned their maize cultivation from the monsoon season to the winter season and they grow rice in the maize fields in monsoon (Chaudhary et al. 2021). The Paoay highlanders of the Philippines plant crops ahead of time to endure frost that occurs between December and February (Launio et al. 2020). A similar practice of planting ahead of time (in the case of rainy season crops) found that it makes the crop withstand the rains (Launio et al. 2020). In Africa, farmers start cultivating with the first rain as they are unsure of how long the rain will last (Sakapaji 2021; Wekesa et al. 2015; Filho et al. 2022) and crops are weeded earlier than in the past (Wekesa et al. 2015).

*Crop rotation and intercropping* are the most dominant adaptation strategies in farming among Indigenous farmers. These are being done as adaptation to drought and floods (Sakapaji 2021; Taremwa et al. 2021; Mensah et al. 2020; Son et al. 2020; Wekesa et al. 2015; Baul and McDonald 2014; Hosen et al. 2020). In India, the Indigenous farmers practise intercropping, also called mixed cropping, in small strips of land to maximise income and food production (Shaffril et al. 2020). Intercropping a primary crop with a legume helps in getting stable yields (Chaudhary et al. 2021; Sakapaji 2021; Son et al. 2020). During periods of drought, intercropping protects the topsoil from wind erosion and during intense rainfall, it absorbs raindrops reducing soil erosion (Mensah et al. 2020; Taremwa et al. 2021). Therefore, intercropping helps diversify income sources, protect soil, reduce pests, and create more jobs for local people (Sakapaji 2021; Mensah et al. 2020; Son et al. 2020) helping farmers in poverty to improve their living conditions (Son et al. 2020). Similarly, crop rotation reduces pests and helps in soil recovery. It boosts yields (Sakapaji 2021; Taremwa et al. 2021; Mensah et al. 2020), reduces reliance on inorganic fertiliser (Mensah et al. 2020) and minimises crop failure (Chaudhary et al. 2021). Crop rotation and intercropping contribute to food security and make the community more resilient to climate emergencies (Baul and McDonald 2014).

*Optimising agricultural methods* is visible with IK holders improving their traditional farming practices. Farmers in Kenya have switched the small traditional hoes with bigger hoes and hire tractors to replace the ox ploughs (Wekesa et al. 2015). It results in higher yields as the deep ploughing allows better aeration and higher water holding capacity (Wekesa et al. 2015). Indigenous people of Baojiatun in China fill their rice fields with river water in advance to protect the crop from hail before hailstorms in spring and summer (Li and Han 2022). Farmers prefer using early maturing crop varieties to harvest before the rains retreat (Siggia et al. 2016; Rai et al. 2022). Research around the world has shown that such flexibility enhances the adaptive capacity and resilience of communities (Sakapaji 2021). In addition, to get organic manure, farmers form alliances with pastoralists and work closely exchanging crop residues for the herders’ livestock to get organic manure (Musa and Umar 2017; Wekesa et al. 2015), which are examples of locally adapted community solutions to climate variability.

*Traditional storage techniques* are a widely prevalent age-old practice among Indigenous communities as they stand highly vulnerable to climate variabilities because of their lack of resources (Rao and Patil 2013; Son et al. 2020; Vargas 2019). Farmers in Haiti, for instance, resort to storing of the high-market value crop like banana (Sharma and Kaushik 2015). Tuvalu has the practice of storing food in advance before natural disasters (Sharma and Kaushik 2015). The pastoral communities of Sikkim in India and Mongolia stock up on food and fodder for the winter season (Ingty 2017: Tugjamba et al. 2021). Egyptians bury grain in the sand and building granary rooms in houses have proven to be effective in preparing farmers for emergencies (Sharma and Kaushik 2015). Kisumu County in Kenya and Eastern Cape Province in South Africa preserve grains by hanging cobs in smoky areas or mixing grains with ash and storing them in a pot sealed with cow dung to shield them from weevils and improving germination rates (Apraku et al. 2021). Indigenous Himalayan communities in India use cow dung and cow urine to protect seeds and grain from pests and diseases (Rana et al. 2019). Kilifi County in Kenya store their harvests for the future by sun drying tubers and grains. The dried grains are stored in a traditional store called a ‘*lutsaga’* which is built above the cooking stove to protect it from pests (Mwaniki and Stevenson 2017). In India, farmers keep partially opened match boxes in storage bins with the phosphorous pasted on the box to keep pests away (Rana et al. 2019) and such traditional storage techniques are effective adaptation strategies helping build community resilience.

Therefore, in farming, IK-driven strategies to adaptation is widely common which includes using resilient crop varieties, adjusting planting schedules, crop rotation and intercropping, and improving on their traditional farming methods to adapt to the impacts of extreme weather events. Traditional storage methods using natural preservatives and building specialised storage facilities are also found to ensure food security during climatic shocks. These practices collectively foster climate change adaptation and resilience by enhancing the adaptive capacities of Indigenous communities using IK.

#### Use of IK in Non-agriculture Sectors

In non-agriculture sectors, IK is utilised for climate change adaptation and resilience by communities of hunters in the Arctic, herders, and fishers, and in water resource management, using plants for medicine, and in the diversification of income sources. This section explores these applications in turn.

*Hunters* in the Arctic use several strategies to adapt to the changing climate which mainly involves the changing ice and snow conditions that is shortening the hunting season (Huntington et al. 2017). In Alaska, hunters are adapting to it by modifying their seasonal hunting schedules, hunting different species, exploring new hunting areas, and using alternative travel routes and transportation methods like all-terrain vehicle (ATV), boats, and snowmachines (Pearce et al. 2015; Huntington et al. 2017), in addition to reading weather and ice conditions before and during travel (Pearce et al. 2015). There are also food-sharing networks established in Canada to provide country foods to community members despite subsistence challenges (Fawcett et al. 2018), community-sponsored hunts are organised to increase access to country foods (Fawcett et al. 2018), and inter-community trade is also initiated to address disparities in food availability (Pearce et al. 2015). Such innovative use of IK greatly enhances the resilience to climate change among the Inuit in the Arctic (Pearce et al. 2015).

*Herders* employ a range of adaptation methods. Migration and transhumance are common adaptation measures adopted by Indigenous pastoral communities for generations to adapt to climate variability (Granderson 2017; Matos et al. 2021; Shaffril et al. 2020;; Sakapaji 2021; Tsosie 2019). Migration to drier areas with water and pasture has always been a common adaptation method for generations (Shaffril et al. 2020; 2017; Sakapaji 2021). Transhumance is seasonal movement of livestock and people among pastoral communities between fixed summer and winter pastures (Fernández-Giménez et al. 2021). It is carried out in response to drought, famines, and land degradation in Africa (Ingty 2017), Vanuatu (Granderson, 2017), India, (Ingty 2017), China (Ingty 2017) and many other parts of the world (Matos et al. 2021; Shaffril et al. 2020). In addition, climate variability (Legide et al. 2024) and seasonal food shortages in Africa (Amare 2018) has influenced transhumance immensely. In the High Atlas Mountains of Morocco, herders practice transhumance to reduce supplementary feeding costs by allowing grazing on natural vegetation throughout the year (Fernández-Giménez et al. 2021). However, for the nomads in Mongolia, traditional seasonal migration is becoming more limited due to water scarcity. Young herders, therefore, reduce movement by staying closer to mobile networks and schools (Tugjamba et al. 2021). Livestock are sold during climate emergencies like drought in Nepal (Shaffril et al. 2020), and harsh winters in Mongolia (Tugjamba et al. 2021). The Fulani herders in the Sahel adapt to water and pasture scarcity (Zougmoré et al. 2023) by diversifying sources of feed by pruning tree branches and leaves, using crop residue, and relying on grass sprouts in valleys from dewfall (Napogbong et al. 2021). Nomads in the Eastern Cape Province of South Africa have transitioned to more resilient cattle breeds, and slaughter the weak for food (Legide et al. 2024). In the middle-hills of Nepal, big livestock like buffalo and cattle are replaced by goats which require lesser fodder and water (Baul and McDonald 2014). In summary, migration, both seasonal and permanent, along with changes in feedstocks and farmed animals are critical adaptation strategies, enhancing subsistence security and contributing directly in building the resilience of Indigenous herding communities.

*Fishers* use IK to develop tools and techniques for fishing like in northeast India where it has proven to be key survival tactics (Inaotombi and Mahanta 2018). In the absence of basic navigation tools, the head fishers use his local knowledge and experience to identify fishing points with great accuracy, thus, displaying the efficacy of IK (Mangada 2021). Fishing techniques has also evolved over time. In the Philippines, the art of making traditional bamboo fish traps and homemade goggles is diminishing with fishers using PET bottles to make traps and using commercial snorkelling masks (Maliao et al. 2022). Such innovations prove the flexibility of IK of fishers which enhances their adaptability and resilience (Maliao et al. 2022). Rwanda fishers practice controlled fishing by harvesting only matured fish from identified spots, which helps in the biological conservation of fish species (Taremwa et al. 2021). Influenced by certain local belief system, other communities have similar eco-friendly fishing practices in certain water surfaces such as the Bajo fishing community in Indonesia (Wani and Ariana 2018), and avoiding fishing on Tuesdays and Fridays in the Philippines (Maliao et al. 2022). As such, fishers employ sustainable techniques and innovative tools building on IK for better adaptation which directly helps in building community resilience.

*Water resource management* in coastal communities involves using IK to address the critical importance of accessing fresh water for human habitation following climatic disasters or rising sea levels. Pacific Island countries have developed various water conservation techniques ranging from Indigenous methods like pond fields, irrigation, and terracing to unique island-specific approaches (Nunn et al. 2024). For instance, Oneisomw Islanders have reopened and cleaned traditional inland wells, mimicking their ancestors’ actions to ensure water quality and supply (Nunn et al. 2024). During droughts, coastal spring water is used by digging wells for washing and swimming, saving water in tanks for drinking and cooking (Rarai et al. 2022), which is seen as an adaptation strategy for sustainable water resource management. IK also play a significant role in preservation of water resources. The Mirriwong people in Australia strictly follow cultural beliefs in collecting water during certain periods (Shaffril et al. 2020) and locals in Zambia refrain from felling trees around some wetlands and river systems (Makondo and Thomas 2018), and avoid building piggeries near water sources in Vanuatu (Granderson 2017; Shaffril et al. 2020). The use of IK in the conservation of water resources form effective adaptation strategies directly contributing to community resilience against the impacts of climate change.

*Using plants for medicine* is common in Indigenous communities (Rana et al. 2019; Kahlon and Singh 2021; Sakapaji 2021; Apraku et al. 2021). In hilltop communities of Odisha in India, Indigenous medicine men called Guniyas use a wide range of plant components to treat various illnesses including respiratory issues, digestive problems, and skin ailments (Kahlon and Singh 2021). Similarly, the Himalayan communities of Himachal in India, use plant leaves to treat skin diseases of animals and humans (Rana et al. 2019). They feed domestic animals with mustard oil to build resistance against diseases and heat stress besides treating worms, bloating, stomach-ache, haemorrhagic disease, and scabies with various plants (Rana et al. 2019). Zambian farmers use a toxic concoction of medicinal extracts from locally available trees like Neem, Peri-Peri, and Sausage, and spray it on the maize crop to kill armyworms and other pests (Sakapaji 2021). The Backan Indigenous people in Vietnam use local medicinal plants and herbs as a coping mechanism to hot weather by drinking it or using it in taking baths, besides treating illnesses like fever and headaches (Manh and Ahmad 2021). In summary, Indigenous people use IK and make use of plants for various medicinal uses for personal health and that of domestic animals and to kill pests and make their crops more adaptable to climatic conditions which helps in adaptation, building their resilience to climate change.

*Income diversification* is critical for sustenance of Indigenous farming communities (Sakapaji 2022; Son et al. 2020; Vargas et al. 2019; Kupika et al. 2019) to outlive climatic uncertainties (Chaudhary et al. 2021; Legide et al. 2024) and to spread risk (Ingty 2017). It is common in many Asia-Pacific countries (Shaffril et al. 2020) especially engaging in waged labour works (Chaudhary et al. 2021; Kaganzi et al. 2021), and non-farm activities (Landicho et al. 2016) like selling firewood and small businesses (Kaganzi et al. 2021). Bangladeshis resort to their traditional practices of rearing ducks and fall back to cottage industries like weaving mats (Sakapaji 2022), which is similar to Zimbabwe (Kupika et al. 2019) and South Africa (Jiri et al. 2015). Flooded lands are converted into fishponds in Bangladesh (Sakapaji 2022) and Rwanda (Taremwa et al. 2021) and use of age-old floating bed gardening to grow vegetable (Sakapaji 2022). In India, while families rely on the household leader for income, other members have overtime, adapted to taking up other income-generating jobs (Ingty 2017; Shaffril et al. 2020). While the use of traditional medicine is prevalent that reduces medicine costs and build resilience (Son et al. 2020), there are also illegal harvests like poaching, hunting, and fishing during natural calamities (Kupika et al. 2019). In summary, diversifying income sources through non-farming activities helps sustain livelihoods helping Indigenous through local adaptation and building community resilience across various non-agriculture sectors.

### What could be done to Maximise the Use of IK Towards Enhancing Climate Adaptation and Resilience?

Maximising the benefits of using IK towards enhancing climate adaptation and resilience is essential for sustainability of Indigenous communities in the face of a changing climate. Two dominant solutions for harnessing the potential of IK emerge from the 71 papers. The first involves the need for more research and bridging^1^ it with scientific knowledge. The second embrace a range of solutions including mainstreaming IK in climate policy through collaborative approaches. These solution options are addressed in turn.

#### The need for more Research and Bridging it with Scientific Knowledge

A prominent solution to maximise the benefits of using IK in building climate adaptation and resilience as outlined by all the 71 papers is the need for more research in IK and practices with a focus on diverse community types impacted by climate change. Indigenous people respond as a group and are very effective as organised communities in responding to climate variables, which research should focus on understanding that knowledge systems of Indigenous people (Petzold et al. 2020; Shaffril et al. 2020; Inaotombi and Mahanta 2018; Sakapaji 2021; Vargas 2019; Mensah et al. 2020) with a multidisciplinary approach as IK is unique to every geographical location (Kahlon and Singh 2021; Petzold et al. 2020; Shaffril et al. 2020).

In the past 30 years, extensive research has been done on IK but the focus in relation to climate change and adaptive capacity building is a recent phenomenon (Granderson 2017). In the past, science always took priority over IK in research and development (Jiri et al. 2015). However, the need for bridging it with scientific knowledge has become more urgent because Indigenous community can no longer rely solely on IK (Launio et al. 2020) or on scientific knowledge (Filho et al. 2022) considering unpredictable changes brought forth by climate change (Launio et al. 2020; Nunn et al. 2024). Therefore, bridging IK and scientific knowledge systems will complement each other (Legide et al. 2024; Ingty 2017; Makondo and Thomas 2018). Although Indigenous people have confidence in IK (Apraku et al. 2021; Mensah et al. 2020), overwhelming focus on the technical aspects leads to a lack of research on climate adaptation methods of Indigenous communities (Mangada, 2021; Sakapaji 2021; Vargas 2019; Inaotombi and Mahanta 2018). More research should be conducted to explore the ecological values of IK and its role in climate change adaptation and resilience (Mangada 2021; Sakapaji 2022; Sakapaji 2021; Tugjamba et al. 2021) in under-researched areas (Kupika et al. 2019), underrepresented regions (Petzold et al. 2020) like the Asia-Pacific territories where 70% of the global Indigenous population resides (Shaffril et al. 2020) and poor communities (Sakapaji 2021; Siggia et al. 2016). A bridged knowledge system can be used to make informed decisions to enhance agricultural food security (Kupika et al. 2019), informing policy on best practices to build adaptative capacity (Kupika et al. 2019), to identify best management approaches (Li and Han 2022), verify models and climate scenarios (Mangada 2021), and it helps in reducing losses due to climate events (Mangada, 2021). Bridging IK and scientific knowledge becomes inevitable in agriculture for effective adaptation, resilience building, and sustainability (Sakapaji 2022).

Future research should focus on exploring how Indigenous communities can influence adaptation policy to provide a more comprehensive understanding of the existing research landscape (Petzold et al. 2020). A bridged knowledge system will provide diverse approaches to allow Indigenous communities to select options that best align with their socio-cultural, political, and ecological contexts (Rarai et al. 2022; Tugjamba et al. 2021; Theodory 2021). For this bridging to be effective, it is necessary to first determine the conditions under which the bridged knowledge systems are mutually consistent and compatible (Sakapaji 2022; Legide et al. 2024). It is also necessary to harmonise the top-down standardisation approaches of governments to the bottom-up approaches of Indigenous communities (Sakapaji 2022; Inaotombi and Mahanta 2018). It has been suggested that there seems to be too much focus on simply documenting IK rather than incorporating it into policy (de Scally and Doberstein 2022) and the bridging of IK and scientific knowledge is still limited in the science-policy process (Filho et al. 2023). Scientific knowledge should accommodate IK in mainstreaming climate change adaptation efforts (Inaotombi and Mahanta 2018; Kupika et al. 2019) and the need for it has been acknowledged by the UN system (Kupika et al. 2019; Inaotombi and Mahanta 2018) including the IPCC (Vargas 2019; Rarai et al. 2022) and the scientific community (Makondo and Thomas 2018; Ingty 2017; Nunn et al. 2024).

Finally, the 71 papers implicitly draw on the research approach required to facilitate the bridging of IK and scientific knowledge. This enquiry leads to literature, away from the suite of 71 papers but crucial for bridging IK and scientific knowledge, to approaches like Two-Eyed Seeing which was introduced by elders of the Mi’kmaq Indigenous tribe in Canada in 2004, Albert Marshall and Murdena Marshall (Wright et al. 2019). Bartlett et al. (2012) in a paper co-authoring the two Mi’kmaq elders, explains Two-Eyed Seeing as “learning to see from one eye with the strengths of Indigenous knowledges and ways of knowing, and from the other eye with the strengths of Western knowledges and ways of knowing, and to using both these eyes together, for the benefit of all” (Bartlett et al. 2012, p. 335). The approach attempts to address the concern noted by Nadasdy (1999, p. 15) that “the goal of knowledge-integration forces TEK researchers to compartmentalise and distil Aboriginal people’s beliefs, values, and experiences according to external criteria of relevance, seriously distorting them in the process.” Two-Eyed Seeing emphasizes weaving of perspectives with both knowledge systems having equal importance where researchers are encouraged to learn weaving back and forth between Indigenous and Western ontologies, epistemologies, and methodologies (Bartlett et al. 2012). Two-Eyed Seeing approach can help in bridging the gap of understanding by bringing together Indigenous and Western worldviews in a collaborative and equitable approach to research (Bartlett et al. 2012). Similarly, other culturally appropriate approaches include Knowledge Weaving/Braiding, and Bridging Knowledge Systems (Stirling et al. 2023).

#### Other Solutions

Across the 71 papers, a wide range of solutions for maximising the use of IK in enhancing climate adaptation and resilience were suggested. These included mainstreaming IK, new approaches to development, involving Indigenous communities in policy, investing in agriculture, empowering governance institutions, awareness creation, intergenerational transmission of IK, and using some other specific innovative approaches. These are discussed in turn.

*Mainstreaming IK* can involve multiple dimensions and engage systematic approaches. The first step towards mainstreaming IK is to begin by documenting it (Clissold et al. 2023; Filho et al. 2023) as IK plays a crucial role in shaping human perceptions and developing adaptation strategies (Chaudhary et al. 2021; Filho et al. 2022; Granderson 2017; Hiwasaki et al. 2015). Effective Indigenous practices can be identified, documented and incorporated into formal adaptation strategies and made available to policymakers, planners, and local communities (Filho et al. 2022; Mwaniki and Stevenson 2017; Nunn et al. 2024) in developing sustainable alternatives (Amare 2018). Zougmoré et al. (2023) posit that having an inventory of the most efficient and successful cases of IK-based adaptation will help inform policies.

*New approaches to development* should be grounded in the principles of deep ecology, as in IK, and it becomes necessary to embrace development with the same principles to effectively cope with a changing climate (Li and Han 2022; Hosen et al. 2020). Climate change adaptation should begin with a deeper understanding of Indigenous people’s perspectives, their realities of climate change, and their Indigenous best practices (Musa and Umar 2017; Son et al. 2020; Guodaar et al. 2021). Policies for developing agriculture and natural resources management should focus on specific social and economic dimensions relevant to the Indigenous communities (Son et al. 2020; Vargas 2019). Scientists and policymakers should lead efforts to bridge IK systems into scientific frameworks. This approach can enhance the utilisation and management of agro-biodiversity and develop more effective strategies to cope with the risks of adverse climatic conditions (Baul and McDonald 2014). The development of policy and regulatory guidelines should be done with a focus on sustainability (Rao and Patil 2013; Datta and Kairy 2024) particularly at the local and community level (Sakapaji 2022; Wekesa et al. 2015; Hosen et al. 2020) for better adaptation and resilience (Wekesa et al. 2015; Inaotombi and Mahanta 2018; Amare 2018). In developing countries, adaptation strategies tend to focus mostly on large scale infrastructure, but should instead focus on local and Indigenous initiatives as they are environment friendly (Amare 2018), cheaper, more sustainable, and easy to up-scale innovations to build adaptation and resilience (Wekesa et al. 2015; Inaotombi and Mahanta 2018; Amare 2018). For Levubu and Nwanedi Indigenous communities in South Africa which are solely reliant on IK systems without any capacity to use scientific knowledge, it is crucial to get support from national institutions and municipal authorities to develop a comprehensive set of policies to allow climate change adaption in the natural environment because existing legislation fail to highlight the role played by IK in climate adaptation (Kom et al. 2023).

*Involving Indigenous communities in climate-related policy making* is crucial to capitalise on IK in building adaptation and resilience to climate change. It requires an inclusive approach to decision-making, planning, and management of climate policies (Inaotombi and Mahanta 2018; Mangada 2021; Sakapaji 2022; Tsosie 2019; Vargas 2019; Wekesa et al. 2015) and to shift the focus to a bottom-up approach (Inaotombi and Mahanta 2018; Mangada 2021; Mensah et al. 2020; Amare 2018) and in engaging multiple stakeholders (Mangada 2021; Amare 2018). Hooli (2016, p. 698) contend that it is “very difficult or even impossible” to find sustainable solutions to local socio-ecological challenges without the full participation of local stakeholders. Datta and Kairy (2024) suggest restructuring adaptation strategies for Indigenous people is critical to address the distinct vulnerabilities and cultural context which is only possible by involving Indigenous people in the process of drafting policy. Effective Indigenous practices can be identified and documented through collaboration with local and religious leaders, agriculture extension officers, and scientists and it can then be integrated into formal adaptation strategies and made accessible to policymakers, planners, and communities (Mwaniki and Stevenson 2017). Even at the macro level, including Indigenous people in government programmes will directly translate into better climate services as IK represents local needs more efficiently and policy makers can make more informed decisions (Mangada 2021; Tsosie 2019; Vargas 2019; Wekesa et al. 2015). IK informed policy decisions can play a crucial role in developing sustainable solutions (Kahlon and Singh 2021; Sakapaji 2022; Datta and Kairy 2024). An effective way of including Indigenous people is for the government to work closely with organisations, associations and groups of Indigenous peoples (Kaganzi et al. 2021). As such, it is important that government offices and donor agencies refrain from imposing the nature of development for Indigenous communities and rather engage them to draft developmental agendas and policies when it comes to building adaptation and resilience to climate change (Mangada 2021).

*Investment in agriculture* will directly help in capitalising on IK as agriculture is the main occupation in many Indigenous communities (Zvobgo et al. 2023; Nkomwa et al. 2014). Finding effective and sustainable solutions in the agriculture sector will enhance adaptive capacity and resilience (Sakapaji 2021; Rai et al. 2022; Negi et al. 2017). New agricultural technologies can be more appropriately designed for diverse contexts when IK is bridged into research and policy (Filho et al. 2022). Many farmers are already combining Indigenous and modern knowledge systems to mitigate climate change risks. Promoting this bridging would avoid maladaptation and enhance livelihoods (Guodaar et al. 2021). For agriculture-reliant Indigenous communities, measures towards enhancing resilience by improving productivity, reducing hunger, and safeguarding the environment is key to their survival (Mensah et al. 2020). Therefore, the agriculture sector should be fully funded to address climate variabilities, particularly at the local level, where people rely mostly on IK to adapt to climate change (Sakapaji 2022; Makondo and Thomas 2018). Investments to support farmers to take up integrated farming (Landicho et al. 2016) with enhanced access to capital to purchase improved seed varieties, buy agrochemicals, irrigate, and implement soil conservation techniques have become necessary (Kaganzi et al. 2021).

*Empowering governance institutions* at the local level is necessary to benefit from IK. Governance capacity at all levels need to be empowered (Sakapaji 2022; Hosen et al. 2020) by investing in human resources, providing access to finance, and improving knowledge management systems (Maliao et al. 2022; Filho et al. 2023) to build adaptation and climate resilience (Sakapaji 2022). Studies in Africa have highlighted the crucial role of local institutions in supporting local adaptation strategies by providing weather forecasts, facilitating information exchange, and managing resource (Legide et al. 2024). Local institutions, grounded in IK, must be integrated with state-level institutions in policy development and implementation to reduce community vulnerability (Ingty 2017) with networks for sound coordination between all stakeholders (Sakapaji 2022; Son et al. 2020; Negi et al. 2017). It is essential for local and national governments to prioritise the development of infrastructure in marginalised communities that depend on natural resources for their survival for climate change adaptation and resilience (Rai et al. 2022). Therefore, policy barriers need to be addressed and capacities of local institutions should be developed to allow bridging of IK into climate change adaptation and disaster risk management policies (Sakapaji 2022).

*Awareness creation* through improved access to information, environmental education, experience sharing among community members, social bonding, and institutional support is crucial for enhancing the use of IK in effective climate change adaptation (Amare 2018; Begg et al. 2023; Rai et al. 2022). Collaboration among key stakeholders—including government agencies, non-government organisations, businesses, and Indigenous people should be strengthened (Begg et al. 2023). Effective communication of climate change issues and knowledge will alert Indigenous people about the impacts of climate change and help identify innovative adaptation strategies (Landicho et al. 2016). Datta and Kairy (2024) found that the prevailing mismanagement and ignorance towards Indigenous communities needs to be addressed. As such, disseminating knowledge and helping Indigenous communities to adopt appropriate mitigation and adaptation measures have become necessary (Rai et al. 2022). Promoting IK among young people and infusing it in formal education systems will be essential (Landicho et al. 2016) for enhancing its applicability and sustainability.

*Intergenerational transmission of IK* to younger knowledge holders has become critical for its conservation and promotion given its significance in climate change adaptation and resilience (Granderson 2017; Tugjamba et al. 2021; Hiwasaki et al. 2015). This warrants IK to be properly documented, archived and researched (Granderson 2017; Hiwasaki et al. 2015; Huntington et al. 2017). Filho et al. (2023) describes the preservation and transmission of IK as one of its biggest challenges. Engaging older knowledge custodians, women, and other vulnerable groups in sharing their expertise with youth is crucial for transferring IK to the younger generation (Granderson 2017; Tugjamba et al. 2021). There is often no mechanism for passing down such knowledge, and young Indigenous people commonly rely on radio or TV for hazard predictions, which lack local specificity (Hiwasaki et al. 2015). It is crucial to preserve this knowledge so the younger generation can use it to strengthen climate change adaptation and build community resilience (Hiwasaki et al. 2015).

*Innovative approaches like Cultural Village and Knowledge Centres* have proven to work well in building climate resilience by empowering Indigenous communities. A Cultural Village in Kenya has become a tourist attraction by allowing local communities to market their culture, increase income sources, and promote social cohesion (Wekesa et al. 2015). Such innovative approaches need to be encouraged for Indigenous communities (Wekesa et al. 2015). The concept of Village Knowledge Centres is an innovative project to help address climate variability and enhance adaptation and resilience (Rao and Patil 2013). In Chennai in South India, the village knowledge centres provide information on local weather forecasting meteorological observatories and crop management practices through internet kiosks (Rao and Patil 2013). In Andhra Pradesh, India, the rural knowledge centres use ICT tools to predict droughts and forecast weather (Rao and Patil 2013). At the community level, ICT tools play a critical role of providing information at the right time apart from understanding future climate risks (Rao and Patil 2013).

Therefore, a multifaceted approach is required to maximise the benefits of using IK towards enhancing climate adaptation and resilience. This includes conducting more research on bridging IK with scientific knowledge with culturally appropriate approaches to research, ensuring that policies mainstream IK through collaborative and inclusive processes, and focusing on specific social and economic dimensions relevant to Indigenous communities. Empowering local governance institutions, raising awareness, promoting intergenerational transmission of IK, and investing in agriculture and innovative approaches such as Cultural Villages and Knowledge Centres are proposed. Embracing such strategies would lead to the development of effective and sustainable solutions that enhance the adaptation and resilience of Indigenous communities against climate change.

## Conclusion

This SLR was conducted to understand how IK contributed to climate change adaptation and resilience. It reviewed 71 papers to show that the topic is multidisciplinary without any authoritative researcher as indicated by the diverse authorship of the final papers. The 71 final papers are geographically skewed towards Africa and Asia with 53 papers (75%) from the two continents.

The study of IK application by Indigenous communities identified three dominant themes: its use in weather prediction, early warning, and disaster preparedness; in agriculture; and in non-agricultural sectors. In Agriculture, IK is used in adapting to disasters and climate uncertainties and the most common strategy is crop rotation, intercropping and use of climate-smart crop varieties. With the changing climate, Indigenous people have also learnt to change their cropping pattern and evolve the Indigenous farming practices. They have learnt to prepare for climate emergencies and use traditional strategies of storing food. In addition to agriculture, IK is used across other sectors to adapt to climate change. They include hunting, migration and transhumance, water management, fishery and income diversifying activities. The use of IK is dominant in using plants for medicine and has also influenced traditional management practices and migration of vulnerable Indigenous communities to escape climate variabilities. One distinct aspect of IK in Indigenous communities all over the world is its rich usage in predicting weather and seasons which serves as earning warning indicators and helps in preparing for disasters or climate extremes. It is also seen that worshipping nature, and religions have influenced IK in distinct ways and laid the foundation of many existing governance, social and management institutions.

To maximise the benefits of using IK towards climate change adaptation and resilience, this research presents a set of solution options under two dominant themes. One, is the need for more research on multiple dimensions of IK and bridging it with scientific knowledge using culturally appropriate approaches to research like Two-Eyed Seeing which emphasizes weaving of perspectives with both knowledge systems having equal importance where researchers are encouraged to learn weaving back and forth between Indigenous and Western ontologies, epistemologies, and methodologies (Bartlett et al. 2012). Two-Eyed Seeing approach can help in bridging the gap of understanding by bringing together Indigenous and Western worldviews in a collaborative and equitable approach to research (Bartlett et al. 2012). Two, is to consider a wide range of solutions including the need to mainstream IK, embracing an approach to development based on the principles of IK, investing in agriculture, empowering governance institutions, awareness creation, prioritising intergenerational transmission of IK, and capitalising from innovative adaptation and resilience building models and initiatives that are already in place.

## Footnotes

1 While most of the 71 identified papers in this study use the word ‘integration’ to describe the incorporation between IK and scientific knowledge, this paper uses the term ‘bridge’ and its derivatives based on extensive literature on ‘Two-Eyed Seeing, Knowledge Weaving/Braiding, and Bridging Knowledge Systems’. As noted by Nadasdy (1999, p. 15) “the project of knowledge-integration also takes for granted existing power relations between Aboriginal people and the state by assuming that traditional knowledge is simply a new form of “data” to be incorporated into already existing management bureaucracies and acted upon by scientists and resource managers”.
